# Radiomics-Assisted Presurgical Prediction for Surgical Portal Vein-Superior Mesenteric Vein Invasion in Pancreatic Ductal Adenocarcinoma

**DOI:** 10.3389/fonc.2020.523543

**Published:** 2020-11-16

**Authors:** Fangming Chen, Yongping Zhou, Xiumin Qi, Rui Zhang, Xin Gao, Wei Xia, Lei Zhang

**Affiliations:** ^1^ Department of Radiology, The Affiliated Wuxi No.2 People’s Hospital of Nanjing Medical University, Wuxi, China; ^2^ Department of Hepatobiliary Surgery, The Affiliated Wuxi No.2 People’s Hospital of Nanjing Medical University, Wuxi, China; ^3^ Department of Pathology, The Affiliated Wuxi No.2 People’s Hospital of Nanjing Medical University, Wuxi, China; ^4^ Suzhou Institute of Biomedical Engineering and Technology, Chinese Academy of Sciences, Suzhou, China

**Keywords:** pancreatic ductal adenocarcinomas, tomography, X-ray computed, radiomics, neoplasm invasion, presurgical evaluation

## Abstract

**Objectives:**

To develop a radiomics signature for predicting surgical portal vein-superior mesenteric vein (PV-SMV) in patients with pancreatic ductal adenocarcinoma (PDAC) and measure the effect of providing the predictions of radiomics signature to radiologists with different diagnostic experiences during imaging interpretation.

**Methods:**

Between February 2008 and June 2020, 146 patients with PDAC in pancreatic head or uncinate process from two institutions were retrospectively included and randomly split into a training (n = 88) and a validation (n =58) cohort. Intraoperative vascular exploration findings were used to identify surgical PV-SMV invasion. Radiomics features were extracted from the portal venous phase CT images. Radiomics signature was built with a linear elastic-net regression model. Area under receiver operating characteristic curve (AUC) of the radiomics signature was calculated. A senior and a junior radiologist independently review CT scans and made the diagnosis for PV-SMV invasion both with and without radiomics score (Radscore) assistance. A 2-sided Pearson’s chi-squared test was conducted to evaluate whether there was a difference in sensitivity, specificity, and accuracy between the radiomics signature and the unassisted radiologists. To assess the incremental value of providing Radscore predictions to the radiologists, we compared the performance between unassisted evaluation and Radscore-assisted evaluation by using the McNemar test.

**Results:**

Numbers of patients identified as presence of surgical PV-SMV invasion were 33 (37.5%) and 19 (32.8%) in the training and validation cohort, respectively. The radiomics signature achieved an AUC of 0.848 (95% confidence interval, 0.724–0.971) in the validation cohort and had a comparable sensitivity, specificity, and accuracy as the senior radiologist in predicting PV-SMV invasion (all *p*-values > 0.05). Providing predictions of radiomics signature increased both radiologists’ sensitivity in identifying PV-SMV invasion, while only the increase of the junior radiologist was significant (63.2 vs 89.5%, *p*-value = 0.025) instead of the senior radiologist (73.7 vs 89.5%, *p*-value = 0.08). Both radiologists’ accuracy had no significant increase when provided radiomics signature assistance (both *p*-values > 0.05).

**Conclusions:**

The radiomics signature can predict surgical PV-SMV invasion in patients with PDAC and may have incremental value to the diagnostic performance of radiologists during imaging interpretation.

## Introduction

Pancreatic ductal adenocarcinoma (PDAC) is a lethal disease, and the five-year survival rate is lower than 8% (1–3). Although surgery remains the only potential chance for a cure, some patients with localized PDAC are not appropriated for upfront surgery or even unresectable due to the involvement of peripancreatic vessels (3–8). Regarding patients with peripancreatic arterial involvement, upfront surgery is known to be associated with a low resection rate and a deteriorated long-term survival (4, 5). In contrast, for patients with isolated peripancreatic venous [portal vein-superior mesenteric vein (PV-SMV)] involvement, long-term survival after extended pancreaticoduodenectomy (PD) with venous resection may be comparable to that achieved by standard PD without venous resection (6–8). However, the determination of surgical PV-SMV invasion is still based on intraoperative diagnosis (9). Preoperative knowledge of PV-SMV invasion status can promote adequate preoperative preparation, which may mitigate the positive margins associated with unplanned PV-SMV resection, decrease unresectable events due to the inexperience of extended PD, and reduce surgery-related complications (6–8, 10).

CT is commonly used to assess possible vascular involvement and plays a significant role in surgical planning (11, 12). Existing radiological classifications for classifying vascular involvement are based on the presence and degree of tumor contact with the vessel (11–14). Several imaging features for evaluating surgical PV-SMV invasion have been introduced, including encasement (>180°) of the tumor-vein relationship (13), deformation, narrowed or stenotic morphology of PV-SMV (14), and the teardrop sign (15). Unfortunately, such above qualitative imaging findings do not accurately classify vascular involvement, especially in peripancreatic venous involvement (16–18). Also, a recent study showed that agreements in the interpretation of tumor-vascular relationships were low among different observers (19).

Radiomics (20, 21) is a data-centric field that processes radiological imaging data by extracting large amounts of quantitative image features, which are subsequently employed to construct novel imaging biomarkers, namely radiomics signature. Previous radiomics studies on PDAC (22–25) have indicated that quantitative image features were closely related to adverse pathological features, therapeutic response, and prognosis after neoadjuvant therapy. However, radiomics research on distinguishing surgical PV-SMV invasion in patients with PDAC is lacking. Furthermore, it is unknown whether radiomics signature could be used as a supplement to radiological classification for PV-SMV invasion.

Therefore, this study aimed to develop a radiomics signature for classifying surgical PV-SMV invasion and to compare performance of the radiomics signature to that of radiologists. In addition, we evaluate changes in diagnostic performance of the radiologists when predictions of radiomics signature are provided during interpretation.

## Materials and Methods

### Patients

This study was approved by the Institutional Review Board of the Affiliated Wuxi No.2 People’s Hospital of Nanjing Medical University and Wuxi No.5 People’s Hospital. The need to obtain informed consent was waived.

**Figure 1:**
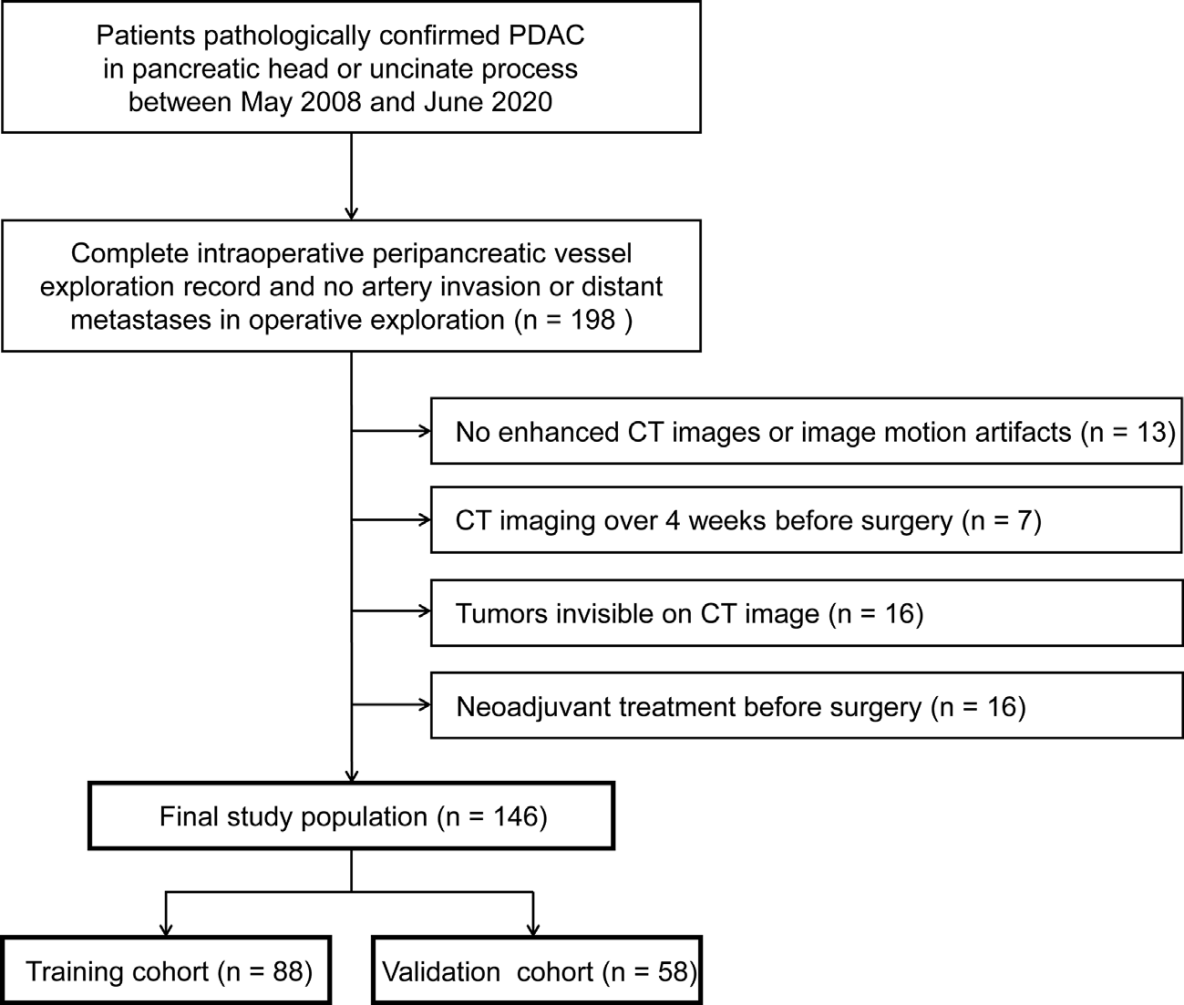
Flow diagram of the study sample. PDAC, pancreatic ductal adenocarcinoma.

From May 2008 to June 2020 in institution 1 (The Affiliated Wuxi No.2 People’s Hospital of Nanjing Medical University) and October 2017 to June 2020 in institution 2 (Wuxi No.5 People’s Hospital), consecutive patients who were treated with surgery and pathologically confirmed PDAC were included. In the institutions, upfront resection, rather than neoadjuvant treatment, was recommended in borderline resectable PDAC with isolated venous involvement. The inclusion criteria were as follows: (1) PDAC in pancreatic head or uncinate process; (2) patients who had a complete intraoperative peripancreatic vessel exploration record; and (3) no artery invasion or distant metastases in intraoperative exploration. The exclusion criteria were as follows: (1) no enhanced CT images or poor image quality; (2) preoperative enhanced CT examination performed more than 4 weeks before the surgery; (3) tumors not visible on CT image; and (4) patients had undergone neoadjuvant therapy before surgery. Ultimately, 146 patients with PDAC were included in this study (**Figure 1**). The patient’s numbers of institutions 1 and 2 were 125 and 21, respectively. Patients were randomly split to a training (n = 88) and a validation cohort (n = 58) according to a ratio of 3:2 using stratified random sampling.

### Definition of Surgical PV-SMV Invasion

In this study, surgical PV-SMV invasion status in PDAC was determined by the findings of intraoperative exploration. Intraoperative appearances of the interface between tumor and vascular were classified as the following types (8, 9): (1) no adherence; (2) adhering but separable; and (3) inseparable. Type 1 or 2 was defined as absence of surgical PV-SMV invasion; type 3 was defined as presence of surgical PV-SMV invasion. All the above procedures were performed by a team of surgeons with at least 10 years of experience (approximately 50 pancreatectomy surgeries annually per surgeon).

### Imaging Techniques

Multiphasic CT was performed by following a pancreatic protocol included unenhanced and contrast-enhanced dual-phasic imaging of the pancreatic parenchymal phase (40–50s) and portal venous phases (65–70s). Images were reconstructed at submillimeter (0.5–1.0mm) thickness in the axial for pancreatic parenchymal and portal venous phase images. Multi-planar reformation and maximal intensity projection reconstructed images of vascular structures were routinely created by radiology technologists and were sent to the Picture Archiving and Communication System (PACS) for interpretation. The pancreatic parenchymal phase produces optimal visual contrast differences between the enhanced pancreatic parenchyma and the tumor; the portal venous phase allows for better evaluation of PV-SMV since the portomesenteric venous system is well enhanced. CT scanners and detailed CT parameters are provided in the **Appendix E1**.

### Tumor Segmentation, Feature Extraction, and Radiomics Signature Building

Tumor segmentation was performed using the ITK-SNAP 3.8.0 (http://www.itksnap.org). A radiologist (FM Chen, 12-year experience in abdominal imaging) selected the slice with the maximum tumor-vein contact on the portal venous phase images and delineated the tumor. The pancreatic parenchymal phase was used to aid determination of tumor boundaries. Another radiologist (B Li, 13-year experience in abdominal imaging) delineated the tumor on a randomly selected cohort of 50 patients following the same procedure.

Image preprocessing and feature extraction were performed using pyradiomics (Version 2.1, https://pyradiomics.readthedocs.io/en/latest/index.html) (21). Images were resampled to a pixel spacing of 1×1 mm^2^. Intensities were discretized with a fixed bin-width of 25 Hounsfield units. Features in 3 categories were extracted from the original images, included 9 shape-based features, 18 first-order features, and 86 grey-level-matrix-based features. The first-order features and grey-level-matrix-based features were additionally extracted from different image transformations, including four wavelet decompositions and five Laplacian of Gaussian filters (sigma = 1.0, 2.0, 3.0, 4.0, and 5.0 mm). A total of 869 features were extracted (detailed in **Appendix E2** and **Table S1**). Each feature value was normalized by the z-score method, which consisted of subtracting the mean value of feature and dividing by the standard deviation of the feature.

The radiomics features were calculated for each radiologist’s delineation, and the intraclass correlation coefficient (ICC) of each feature was calculated to test the inter-observer reproducibility. The features with an ICC≥0.8 were proceeded to subsequent analyses. The linear elastic-net regression was used for feature selection and radiomics signature building. In the hyper-parameter tuning of linear elastic-net regression (26), the α penalty was set to 0.5 following a grid search with the penalty parameter λ determined by 5-fold cross-validation. The built radiomics signature provides a mathematical formula that predicts PV-SMV invasion by using the selected radiomics features with the equation:

$$\hat{y}=\beta_{1}X_{1}+\beta_{2}X_{2}+\cdots +\beta_{i}X_{i}+b$$

In which $\hat{y}$ is the radiomics score (Radscore), b is the intercept, β_i_ is the coefficient of the feature i, and X_i_ is the value of the feature i.

### Radiologists Assessment

There were two readings performed in this study. First, blinded to clinical information, surgical findings, and the Radscore but knowing patients were diagnosed as PDAC, two radiologists (L Zhang, a senior radiologist with 18-year experience in abdominal imaging, and SL. Zhang, a junior radiologist with 5-year experience in abdominal imaging) independently reviewed the CT scans of all patients. Then, after a washout period of 2 weeks, the two radiologists independently reviewed the CT scans of patients in the validation cohort with Radscore assistance but still blinded to the clinical information and surgical findings. For each reading, radiologists were asked to document the following three imaging features:

(1) Tumor was in contact with the PV-SMV for more than 180° (13); (2) PV-SMV blood vessel morphology was deformed, narrowed, or stenotic (14); and (3) PV-SMV was deformed, demonstrating a teardrop shape on axial image (15).

In the first reading, PV-SMV invasion was determined as presence when any one of the above imaging features was present.

**Figure 2:**
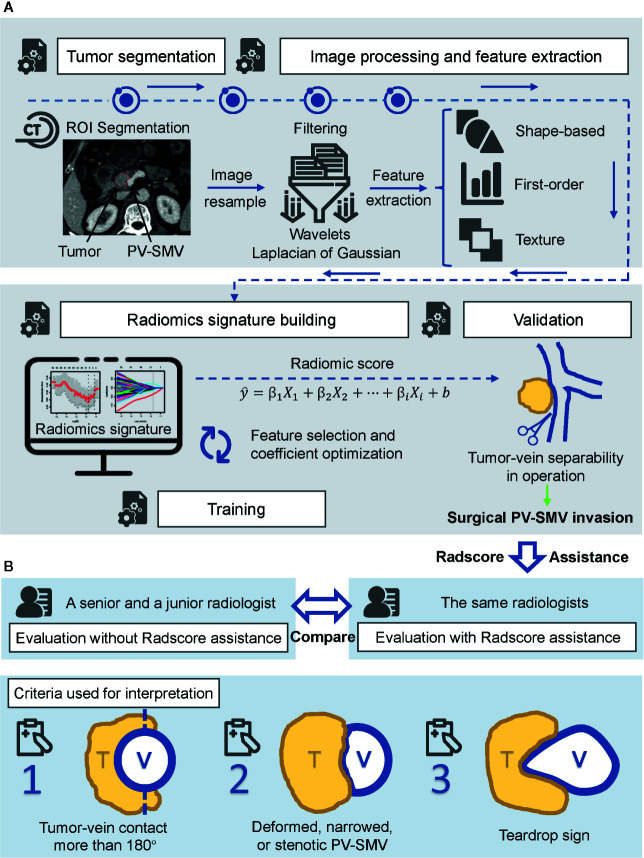
Flowchart of the study. **(A)** Radiomics workflow, including ROI segmentation, feature extraction, radiomics signature construction, and validation. **(B)** Radiologists assessment. Performance of each radiologist between unassisted evaluation and Radscore-assisted evaluation was compared in the validation cohort. Radscore, radiomics score.

In the second reading, the radiologists had been informed of the radiomics-signature-predicted probability of PV-SMV invasion before documenting the imaging features; PV-SMV invasion was also determined as presence when any one of the above imaging features was present. Flowchart of the study is shown in **Figure 2**.

### Statistical Analysis

In statistical tests of clinical features, the Mann-Whitney test was used for continuous variables, and Fisher’s exact test was used for categorical variables. The performance of the radiomics signature was assessed using area under receiver operating characteristic curve (AUC). The optimal cutoff value of Radscore was selected by maximizing the Youden index (sensitivity + specificity − 1). The sensitivity, specificity, and accuracy of the radiomics signature and the radiologists (with or without Radscore assistance) were also reported. A two-sided Pearson’s chi-squared test was used to compare the performance of the Radscore to that of the radiologists. To assess the incremental value of providing Radscore predictions to the radiologists, we compared the performance measures between unassisted evaluation and Radscore-assisted evaluation by using the McNemar test. All comparisons were performed in the validation cohort. Interobserver agreement between the senior and the junior radiologists (with and without Radscore assistance) was evaluated using the Kappa (κ) test: κ-value of 0.2 to 0.4, fair agreement; κ-value of 0.4 to 0.6, moderate agreement; κ-value of 0.6 to 0.8, substantial agreement; κ-value greater than 0.8, almost perfect agreement.

R (version 3.5.1) statistical software was used for statistical analysis in this study. Glmnet R package was used to perform the linear elastic-net regression. Bilateral *p*-value < 0.05 was considered statistically significant.

## Results

### Patient Characteristics

The characteristics of patients are summarized in **Table 1**. No significant differences were found in clinical and surgical factors between the training and validation cohorts. A total of 33 (37.5%) patients in the training cohort and 19 (32.8%) patients in the validation cohort were confirmed PV-SMV invasion in surgical exploration. For these patients, extended surgery was performed when reconstructable PV-SMV involvement can be achieved, otherwise, palliative surgery was performed.

**Table 1:** Patient characteristics.

Characteristics	Training cohort(N = 88), %	Validation cohort(N = 58), %	*p*-value
Age^†^, years	65 (59–71)	67 (59–72)	0.387
Sex			0.903
F	34 (38.6)	21 (36.2)	
M	54 (61.4)	37 (63.8)	
ADL			0.593
Grade 1	18 (20.5)	9 (15.5)	
> Grade 1	70 (79.5)	49 (84.5)	
Weight loss			0.874
Yes	34 (38.6)	24 (41.4)	
No	54 (61.4)	34 (58.6)	
Jaundice			0.746
Yes	37 (42.0)	22 (37.9)	
No	51 (58.0)	36 (62.1)	
Pain			0.567
Yes	54 (61.4)	32 (55.2)	
No	34 (38.6)	26 (44.8)	
Pancreatitis			0.819
Yes	26 (29.5)	19 (32.8)	
No	62 (70.5)	39 (67.2)	
CA 19–9^†^, U/mL	890.5(23–1718.1)	858.5(29–1450.8)	0.885
Clinical T stage			0.659
T1c	16 (18.2)	9 (15.5)	
T2	61 (69.3)	44 (75.9)	
T3	11 (12.5)	5 (8.62)	
Tumor size on CT^†^, cm	2.80 (2.20–3.60)	2.75 (2.30–3.40)	0.946
Length of tumor-vein contact on CT^†^, cm	2.04 (1.59–2.54)	1.79 (1.08–2.43)	0.099
Tumor differentiation			0.453
Poor	3 (3.41)	0 (0.00)	
Moderate	51 (58.0)	36 (62.1)	
Well	34 (38.6)	22 (37.9)	
Operation			0.898
Standard PD	68 (77.3)	46 (79.3)	
Extend PD	5 (5.68)	2 (3.45)	
Palliative Surgery	15 (17.0)	10 (17.2)	
Surgical PV-SMV invasion			0.683
Yes	33 (37.5)	19 (32.8)	
No	55 (62.5)	39 (67.2)	

ADL, activities of daily living; CA 19–9, carbohydrate antigen 19–9; PV-SMV, portal vein-superior mesenteric vein; PD, pancreaticoduodenectomy. ^†^Values are median (IQR).

### Diagnostic Performance of the Radiomics Signature and the Radiologists

**Figure 3:**
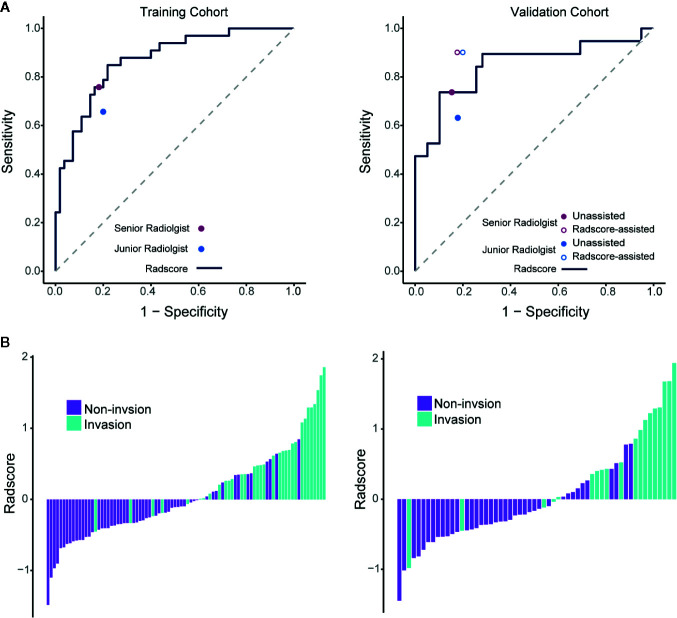
Performance of the radiomics signature with operating points of unassisted and assisted radiologists and the value of Radscores per patient. **(A)** ROC curves of the radiomics signature in the training and validation cohorts. Individual unassisted radiologist (specificity, sensitivity) points are also plotted, where the purple point represents unassisted senior radiologist, and the blue points represents unassisted junior radiologist. In the validation cohort, individual assisted radiologist (specificity, sensitivity) points are also plotted, where the purple circle represents Radscore-assisted senior radiologist, and the blue circle represents Radscore-assisted junior radiologist. **(B)** Radscore (subtraction of the cut-off determined by maximizing the Youden index) per patient in the training and validation cohorts. ROC, Receiver operating characteristic; Radscore, radiomics score.

Among the 869 extracted radiomics features, 751 features with high stability (ICC≥0.8) were identified. The radiomics signature for PV-SMV invasion was developed using the elastic net model (α=0.5, λ=0.174) and retained 10 features, including one morphological feature and 9 texture features (**Table 2**). The ability of the radiomics signature to discriminate PV-SMV invasion was shown to have an AUC of 0.871 [95% confidence interval (CI) 0.795–0.946] in the training cohort and 0.848 (95% CI 0.724–0.971) in the validation cohort (**Figure 3A**). Values of Radscores per patient in the training and validation cohorts are plotted in **Figure 3B**.

**Table 2:** Features in the radiomics signature.

Features	Coefficient
Shape_Sphericity	−0.35592
Log-sigma-2-0-mm-_GLCM_Idn	0.03402
Log-sigma-4-0-mm-_GLSZM_ZonePercentage	−0.00189
Log-sigma-5-0-mm-_GLCM_Imc1	−0.01899
Log-sigma-5-0-mm-_ GLSZM _RunEntropy	0.22822
Log-sigma-5-0-mm-_GLSZM_LargeAreaHighGrayLevelEmphasis	0.02114
Log-sigma-5-0-mm-_GLDM_LowGrayLevelEmphasis	−0.03426
Log-sigma-5-0-mm-_GLDM_SmallDependenceLowGrayLevelEmphasis	−0.13823
Wavelet-HH_GLCM_Correlation	−0.05178
Wavelet-HH_GLSZM_LowGrayLevelZoneEmphasis	−0.07195

Log, Laplacian of Gaussian; GLCM, Gray-level co-occurrence matrices; GLSZM, Gray-level size zone matrix; GLDM, Gray level dependence matrix.

The optimal cutoff value of the Radscore was determined at the level of −0.608. Accordingly, sensitivity, specificity, and accuracy of the radiomics signature for differentiating PV-SMV invasion were determined. There were no significant differences in the performance metrics of the radiomics signature and each radiologist (**Table 3**). The radiomics signature sensitivity (78.9%) for PV-SMV invasion was slightly higher than the radiologists (73.7% of the senior radiologist and 63.2% of the junior radiologist). The Radscore achieved a specificity of 74.4% and an accuracy of 75.9%, while the senior radiologist and the junior radiologist achieved a specificity of 84.6% and 82.1%, and an accuracy of 81.0% and 75.9%, respectively.

**Table 3:** Comparison of the radiomics signature and radiologists on the validation cohort.

Prediction	Sensitivity	*p*-value	Specificity	*p*-value	Accuracy	*p*-value
Senior Radiologist	73.7%	0.70	84.6%	0.26	81.0%	0.24
Junior Radiologist	63.2%	0.28	82.1%	0.41	75.9%	> 0.99
Radiomics signature	78.9%		74.4%		75.9%	

Radscore, radiomics score. A two-sided Pearson’s chi-squared test was used to evaluate whether there was a difference between the radiomics signature and each radiologist (without radiomics signature assistance).

### Incremental Value of Radscore Assistance

**Figure 4:**
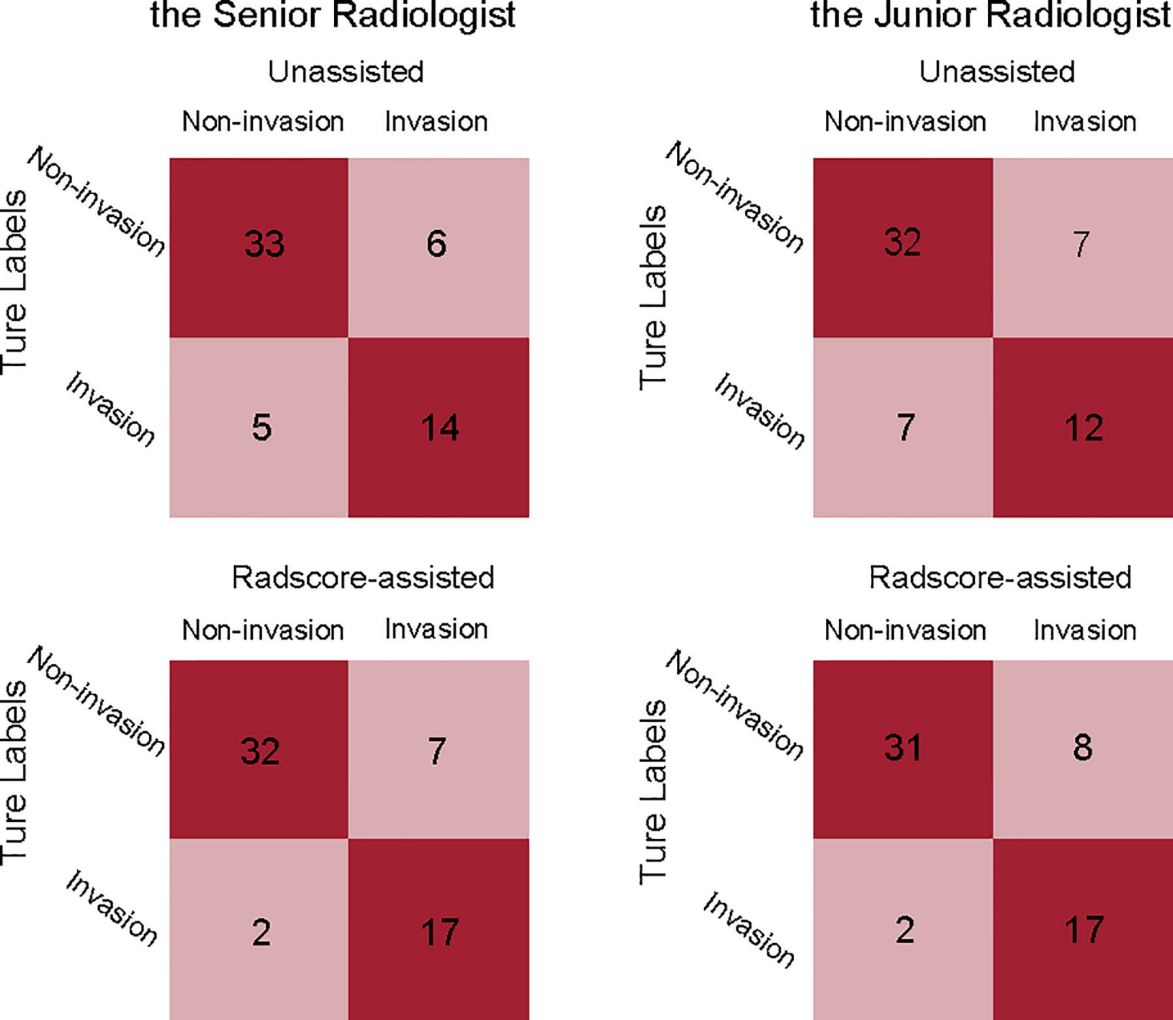
Confusion matrices comparing the true labels and the diagnostic labels. Each plot illustrates performance on the validation cohort. left, the senior radiologist’s unassisted evaluation and Radscore-assisted evaluation; right, the junior radiologist’ unassisted evaluation and Radscore-assisted evaluation. Radscore, radiomics score.

**Figure 5:**
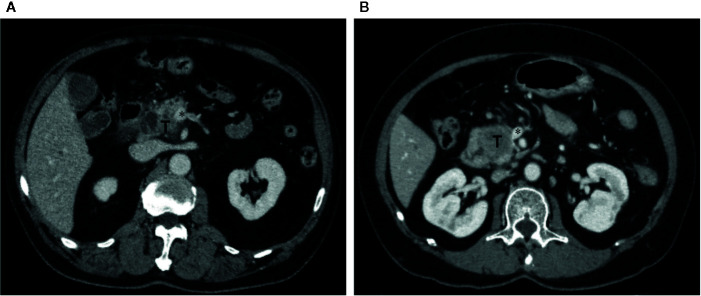
Representative cases which were reclassified after Radscore assistance. **(A)** Axial portal venous phase CT image in a 73-year-old man with PDAC in the pancreatic head. The vein was suspiciously deformed (*). The Radscore prediction gave a high probability of PV-SMV invasion. The interpretation of deformity of the vein between unassisted and Radscore-assisted was discrepant for both radiologists, they assigned PV-SMV invasion category with Radscore assistance. Intraoperative exploration confirmed the diagnosis of PV-SMV invasion. **(B)** Axial portal venous phase CT image in a 66-year-old female with PDAC in the pancreatic head. The vein was interpreted as teardrop shape by the junior radiologist (*) in first the reading. The Radscore prediction gave a low probability of PV-SMV invasion. The vein was interpreted as absence of teardrop shape by the junior radiologist (*) in the second reading with Radscore assistance, and PV-SMV non-invasion category was assigned. Intraoperative exploration confirmed the diagnosis of PV-SMV non-invasion.

Comparison of unassisted and Radscore-assisted performance of each radiologist is illustrated in **Figure 3A** (right), with numerical values presented in **Table 4**. To show the changes of diagnosis after the assistance, confusion matrices of each radiologist are shown in **Figure 4**. When provided Radscore assistance, there was an increase in sensitivity in identifying PV-SMV invasion; for both the junior radiologist (63.2 vs 89.5%, *p*-value = 0.025) and the senior radiologist (73.7 vs 89.5%, *p*-value = 0.08). Even though the radiologist’ specificity was slightly decreased (84.6 vs 82.1% for the senior radiologist and 82.1 vs 79.5% for the junior radiologist) when provided Radscore assistance, the accuracy was slightly increased (81.0 vs 84.5% for the senior radiologist and 75.9 vs 85.8% for the junior radiologist), but neither was significant (all *p*-values > 0.05, **Table 4**). With Radscore assistance, κ-value of inter-rater reliability increased from 0.571 to 0.757 (both *p*-values <0.001). Representative cases which were reclassified after Radscore assistance are shown in **Figure 5**.

**Table 4:** Comparison of unassisted and assisted performance in each radiologist on the validation set.

Prediction	Sensitivity	*p*-value	Specificity	*p*-value	Accuracy	*p*-value
Senior Radiologist						
unassisted	73.7%	0.08	84.6%	0.32	81.0%	0.31
Radscore-assisted	89.5%		82.1%		84.5%	
Junior Radiologist						
unassisted	63.2%	0.025	82.1%	0.70	75.9%	0.24
Radscore-assisted	89.5%		79.5%		82.8%	

Radscore, radiomics score. McNemar test was used to evaluate whether there was a difference between the unassisted and assisted performance of each radiologist.

## Discussion

In this study, we developed a Radscore on presurgical pancreatic enhanced CT in classifying surgical PV-SMV invasion of PDAC in the pancreatic head or uncinate process. In addition, we compared performance between unassisted and Radscore-assisted reviews of radiologists with different diagnostic experiences. Our results demonstrated that the Radscore achieved an AUC 0.848 (95% CI 0.724–0.971) for discriminating PV-SMV involvement and had a comparable diagnostic performance as the senior radiologist. We also found that providing predictions of Radscore to the junior radiologist as a diagnostic aid led to significant improvement in sensitivity for identifying surgical PV-SMV invasion.

Accurate estimation of surgical PV-SMV invasion plays a vital role in the perioperative management of patients with PDAC (11, 12). In this study, prediction-related features consisting of the radiomics signature included one shape-based feature (sphericity) and nine texture features. Among them, sphericity is a measure of roundness of the shape of the tumor region and was an important component for predicting PV-SMV invasion. A previous study (27) reported that unfavorable tumor morphology was highly associated with the presence of peripancreatic vessels involvement. Features related to shape were associated with morphology of the tumor region adjacent to the vein on CT images. Moreover, shape-based features are independent of imaging acquisition parameters and imaging preprocessing techniques, and thus may be highly reproducible. In addition, recent studies (22–25) have suggested that texture features indicating inhomogeneity in imaging are associated with increased intra-tumor heterogeneity of PDAC. The results of this study indicated that some texture features may be closely related to adverse tumor biology in PV-SMV involvement.

In this study, the radiomics signature provided individualized predictions of PV-SMV invasion and the delineation of ROI was easy. Several reasons were explaining why we used 2D ROI instead of 3D ROI. First, the focus of our studies was tumor invasion of PV-SMV, which most likely takes place in the tumor-PV-SMV contact region. Second, delineating 3D ROI slice by slice on the submillimeter-thick images was labor-intensive and may decrease reproducibility. We evaluated inter-reader agreement in delineating 2D ROI and found most features (86.5%) were highly stable. We also compared diagnostic performance of radiomics signature and two radiologists with different diagnostic experiences; even though the radiomics signature did not significantly outperform the junior radiologist, it achieved comparable performance as the senior radiologist. Hence, the proposed radiomics signature combing both morphological and texture parameters was a valuable marker for surgical PV-SMV invasion.

Interestingly, though the interobserver agreement between the radiologists was moderate, their sensitivities were both unsatisfactory. The diagnostic performance of the radiologists was concordant with that of similar studies on CT (16–19); about 20% of cases were false negative according to the qualitative imaging features. Though a recent study reported that endoscopic ultrasound elastography could improve diagnostic performance of vascular invasion in PDAC, the elastography technique was not commonly used for all patients (28). As we knew, image interpretation for PDAC peripancreatic vascular invasion was based on visual assessment of tumor-vein relationship. Vascular morphologic changes on visual assessment, such as vascular deformation, narrowing, or teardrop sign, sometimes were difficult to judge the presence of invasion, this could result in low sensitivity of radiologists in detecting PV-SMV invasion. Concordance to the previous study, pancreatitis-related changes usually blur the contact region between the solid tumor and adjacent vessels; this led to discrepancy in the interpretation of the degree of tumor-vascular contact (abutment vs encasement, <180° vs >180°, respectively) and may have caused the interobserver variability (19).

To examine the effect of the Radscore may have on the interpretation performance of radiologists with different experiences, our study compared unassisted and Radscore-assisted performance of each radiologist in the validation cohort. We found a statistically significant improvement in sensitivity of the junior radiologist (*p*-value=0.025) for discriminating PV-SMV involvement with Radscore assistance and, though no statistically significant increase in accuracy. This was because qualitative features in some cases were relabeled by radiologists with Radscore assistance; as visual assessment has limited capabilities to discern subtle changes. Besides, Radscore assistance also resulted in a higher level of interobserver agreement. Similar findings have been reported in recent deep-learning-assisted diagnosis studies (29, 30). Our results implied that the proposed radiomics signature could be a new imaging marker providing surrogate information for PV-SMV invasion and help to overcome the limitations of subjective visual assessment. To our knowledge, this is the first study to explore providing predictions of the Radscore to assist radiologists in image interpretation of discriminating PV-SMV invasion.

Our study had several limitations. First, as it was a retrospective study, there was an unavoidable selection bias: the study only included patients who had undergone surgical treatment. Second, the study sample was relatively small. Third, we did not examine the relationships between radiological findings and histopathologic vein invasion. As we knew, histopathologic vein invasion is a significant prognostic factor; but the focus of the study is surgical PV-SMV invasion, which may contribute to elaborate preoperative planning of PD, with or without PV-SMV resection and reconstruction. Last, we excluded patients who received neoadjuvant therapy before surgery since several studies have suggested that conventional cross-sectional imaging often failed to identify the extent of the remaining viable tumor (31, 32).

In conclusion, we developed a radiomics signature that achieved comparable performance to radiologists for identifying surgical PV-SMV invasion in patients with PDAC. The radiomics signature could be a new imaging maker and demonstrated incremental value to radiologists in diagnosing surgical PV-SMV invasion.

## Data Availability Statement

All datasets generated for this study are included in the manuscript/**Supplementary Material**.

## Ethics Statement

The studies involving human participants were reviewed and approved by the Ethics Review Committee of the Affiliated Wuxi No. 2 People’s Hospital of Nanjing Medical University and Wuxi No. 5 People’s Hospital. Written informed consent for participation was not required for this study in accordance with the national legislation and the institutional requirements.

## Author Contributions

FC and LZ designed the study. FC, YZ, and WX conducted the experiments. FC, LZ, YZ, and XQ analyzed the data. RZ and XG advised the study and revised the draft. FC wrote the draft. All authors contributed to the article and approved the submitted version.

## Funding

This study is supported by the foundation of Wuxi Municipal Bureau on Science and Technology (CN) (N20192023), the Youth Science Project of Wuxi Health Committee (CN) (Q201727), and the Key Project of Science and Technology Development Foundation of Nanjing Medical University (CN) (2017NJMUZD116).

## Conflict of Interest

The authors declare that the research was conducted in the absence of any commercial or financial relationships that could be construed as a potential conflict of interest.

## Abbreviations

PDAC, Pancreatic ductal adenocarcinoma; PV-SMV, portal vein-superior mesenteric vein; PD, pancreaticoduodenectomy; ICC, intraclass correlation coefficient; Radscore, radiomics score; AUC, area under receiver operating characteristic curve.
