# Supplementary material for: Radiomics-Assisted Presurgical Prediction for Surgical Portal Vein-Superior Mesenteric Vein Invasion in Pancreatic Ductal Adenocarcinoma
Source: Front Oncol. 2020 Nov 16;10:523543. doi: 10.3389/fonc.2020.523543 (PMC7706539; doi:10.3389/fonc.2020.523543)
Supplement: Supplementary file 1 [file Table_1.docx]

***Supplementary materials***

**Appendix E1. CT imaging protocol**

CT examinations were performed using one of four multidetector-row CT scanners, namely Toshiba Aquilion (Toshiba Medical Systems, Tokyo, Japan), Toshiba Aquilion One (Toshiba Medical Systems, Tokyo, Japan), Somatom Definition AS (Siemens Medical Systems, Forchheim, Germany), or Philips Brilliance-128 (Philips Medical Systems, Cleveland, USA). The CT scan parameters were as follows: automatic tube current; tube voltage, 120 kV; rotation time, 0.5 seconds; detector pitch, 0.641–0.828; matrix, 512 × 512; detector collimation, 0.5–1.0 mm × 64; slice thickness/interval, 0.5–1 mm. All patients were fasted for 8 hours before the examination and drank 800–1,000 ml of water 20 to 30 minutes before the scan. The non-ionic contrast agent, Iopromide (Ultravist 370, Bayer, Germany) or Ioversol (Optiray 350, Guebet, France), was injected at an injection flow rate of 3 ml/s. The total amount of contrast agent injection was calculated as 1.5 ml per kilogram of body weight of the patient. The contrast agent was injected via the anterior elbow vein using a high-pressure injector (Stellant, Medrad, USA or Mallinckrodt, Tyco Healthcare, Canada).

**Appendix E2. Feature extraction**

A total of 869 radiomics features including (i) 9 shape-based features, (ii) 14 first-order features (iii) 68 matrix-based features, (iv) 344 wavelet features, and (v) 258 Laplacian of Gaussian transformed features, were extracted. Names of the extracted feature were detailed in **Table S1.** Mathematical definition of these features could be found in https://pyradiomics.readthedocs.io/en/latest/radiomics.html. Most features extracted are in compliance with the definition described in the Imaging Biomarker Standardization Initiative (IBSI) reference manual [1].

**Table S1. Extracted features in this study.**

| **Feature**  **class (*N*)** | **Names** |
| --- | --- |
| Shape  (*N*=9) | Elongation, MajorAxisLength, MaximumDiameter, MeshSurface, MinorAxisLength, Perimeter, PerimeterSurfaceRatio, PixelSurface, and Sphericity |
| First-order features  (N=18) | 10Percentile, 90Percentile, Energy, Entropy, InterquartileRange, Kurtosis,  Maximum, MeanAbsoluteDeviation, Mean,  Median, Minimum, Range, RobustMeanAbsoluteDeviation, RootMeanSquared,  Skewness, TotalEnergy, Uniformity, and Variance |
| GLCM-based  features  (*N*=22) | Autocorrelation, JointAverage, ClusterProminence, ClusterShade, ClusterTendency, Contrast, Correlation, DifferenceAverage, DifferenceEntropy, DifferenceVariance, JointEnergy, JointEntropy, Imc1, Imc2, Idm, Idmn, Id, Idn, InverseVariance, MaximumProbability, SumEntropy, and SumSquares |
| GLRLM-based  features  (*N*=16) | GrayLevelNonUniformity, GrayLevelNonUniformityNormalized, GrayLevelVariance, HighGrayLevelRunEmphasis, LongRunEmphasis,  LongRunHighGrayLevelEmphasis, LongRunLowGrayLevelEmphasis,  LowGrayLevelRunEmphasis, RunEntropy, RunLengthNonUniformity,  RunLengthNonUniformityNormalized, RunPercentage, RunVariance, ShortRunEmphasis, ShortRunHighGrayLevelEmphasis, and ShortRunLowGrayLevelEmphasis |
| GLSZM-based  features  (*N*=16) | GrayLevelNonUniformity, GrayLevelNonUniformityNormalized  GrayLevelVariance, HighGrayLevelZoneEmphasis, LargeAreaEmphasis,  LargeAreaHighGrayLevelEmphasis, LargeAreaLowGrayLevelEmphasis,  LowGrayLevelZoneEmphasis, SizeZoneNonUniformity,  SizeZoneNonUniformityNormalized, SmallAreaEmphasis,  SmallAreaHighGrayLevelEmphasis, SmallAreaLowGrayLevelEmphasis,  ZoneEntropy, ZonePercentage, and ZoneVariance |
| GLDM-based  feature  (*N*=14) | DependenceEntropy, DependenceNonUniformity,  DependenceNonUniformityNormalized, DependenceVariance,  GrayLevelNonUniformity, GrayLevelVariance, HighGrayLevelEmphasis,  LargeDependenceEmphasis, LargeDependenceHighGrayLevelEmphasis,  LargeDependenceLowGrayLevelEmphasis, LowGrayLevelEmphasis,  SmallDependenceEmphasis, SmallDependenceHighGrayLevelEmphasis, and SmallDependenceLowGrayLevelEmphasis |
| Wavelets  (*N*=86*4) | First-order features (N=18) and matrix-based features (N=68) were extracted from four decompositions (Wavelet_LL, Wavelet_LH, Wavelet_HL, Wavelet_HH) after wavelet filter applied to the input image. |
| Laplacian of Gaussian  (*N=*86*5) | First order features (N= 18) and matrix-based features (N=68) were extracted from five derived images (sigma = 1.0, 2.0, 3.0, 4.0, 5.0 mm) after Laplacian of Gaussian filter applied to the input image. |

Note: GLCM, Gray-level co-occurrence matrices; GLRLM, Gray-level run length matrix; GLSZM, Gray-level size zone matrix; GLDM, Gray level dependence matrix.

**References**

[1] Zwanenburg A, Vallières M, Abdalah MA, et al. The Image Biomarker Standardization Initiative: Standardized Quantitative Radiomics for High-Throughput Image-based Phenotyping. Radiology. 2020;295(2):328-338. doi:10.1148/radiol.2020191145
